# *Leishmania*, microbiota and sand fly immunity

**DOI:** 10.1017/S0031182018001014

**Published:** 2018-06-20

**Authors:** Erich Loza Telleria, Andrea Martins-da-Silva, Antonio Jorge Tempone, Yara Maria Traub-Csekö

**Affiliations:** Laboratório de Biologia Molecular de Parasitas e Vetores, Instituto Oswaldo Cruz-Fiocruz, Av. Brasil 4365, Rio de Janeiro, RJ, Brazil

**Keywords:** *Leishmania*, microbiota, vector–microbe interaction, viruses, sand fly

## Abstract

In this review, we explore the state-of-the-art of sand fly relationships with microbiota, viruses and *Leishmania*, with particular emphasis on the vector immune responses. Insect-borne diseases are a major public health problem in the world. Phlebotomine sand flies are proven vectors of several aetiological agents including viruses, bacteria and the trypanosomatid *Leishmania*, which are responsible for diseases such as viral encephalitis, bartonellosis and leishmaniasis, respectively. All metazoans in nature coexist intimately with a community of commensal microorganisms known as microbiota. The microbiota has a fundamental role in the induction, maturation and function of the host immune system, which can modulate host protection from pathogens and infectious diseases. We briefly review viruses of public health importance present in sand flies and revisit studies done on bacterial and fungal gut contents of these vectors. We bring this information into the context of sand fly development and immune responses. We highlight the immunity mechanisms that the insect utilizes to survive the potential threats involved in these interactions and discuss the recently discovered complex interactions among microbiota, sand fly, *Leishmania* and virus. Additionally, some of the alternative control strategies that could benefit from the current knowledge are considered.

## Introduction

Insect-borne diseases are a significant public health problem worldwide. The most important vectors of these diseases are mosquitoes and sand flies and, between these, mosquitoes are the best-studied vectors. Much is known about mosquito interactions with malaria-causing plasmodia and arboviruses (Saraiva *et al.*, 2016). Many aspects of these interactions, including the mosquito immune responses to pathogenic microbes and the role of resident microbiota on the infection and on immune responses of the insects have been reviewed (Clayton *et al.*, 2014; Saraiva *et al.*, 2016). In this review, we discuss some of these aspects in sand flies (Diptera: Psychodidae: Phlebotominae). We will focus mainly on bacteria found in resident microbiota and also on some aspects of the interactions of sand fly vectors with viruses, bacteria and *Leishmania*, with special emphasis on sand fly immune responses.

Sand flies are well-known vectors of leishmaniasis, but they also transmit viruses (Depaquit *et al.*, 2010; Alkan *et al.*, 2013) and bacteria (Herrer and Christensen, 1975; Maroli *et al.*, 2013). The presence of viruses in sand flies has been reported since the middle of last century (reviewed in Tesh and Chaniotis, 1975; Tesh, 1988; Depaquit *et al.*, 2010). Among the viruses transmitted by sand flies, Phleboviruses are considered the most significant, since many of the viruses in this genus are human pathogens capable of causing symptoms varying from short-term fever to meningitis, encephalitis and haemorrhagic fever (Alkan *et al.*, 2013). Sand flies from the *Lutzomyia* genus have been incriminated as vectors of the bacteria causing bartonellosis, also known as Carrion's disease, Oroya fever or ‘verruga peruana’ (Schultz, 1968; Cohnstaedt *et al.*, 2011; Battisti *et al.*, 2015). This disease is characterized by symptoms such as fever and hemolytic anaemia and in a later phase can produce nodular skin lesions (reviewed in Maguina *et al.*, 2009). Little is known about the molecular interactions of sand flies with viruses and bacteria. The following review will present the progress of the field in addressing the responses of the sand fly to the diverse agents it propagates, with a specific emphasis on the expanding understanding of how these responses may be modulated by the insect's microbiota.

Leishmaniases are the most important illnesses transmitted by phlebotomine sand flies. These multi-spectrum diseases present symptoms that vary from ulcerative skin lesions to mucosal deforming lesions (tegumentary leishmaniasis) or liver and spleen hypertrophy (visceral leishmaniasis). Protozoans from the genus *Leishmania* (Trypanosomatida: Trypanosomatidae) are the aetiological agents of leishmaniases. Around 20 *Leishmania* species are known to be pathogenic to humans (Maroli *et al.*, 2013). These digenetic parasites need two hosts to complete their life cycle: one of them a sand fly, while the other can be a human or another mammal. As an exception to sand fly transmission, in Australia biting midges (Diptera: Ceratopogonidae) were implicated in the transmission of the autoctone *Leishmania enriettii* (Dougall *et al.*, 2011; Seblova *et al.*, 2015).

A detailed understanding of how pathogens interact with their vectors and the resident microbes can lead to the discovery of new tools to block disease transmission. New microbe-based blocking tools have been discovered for the mosquitos that transmit malaria (Wang and Jacobs-Lorena, 2013) and there is an excellent evidence that *Wolbachia* endosymbionts can be used as a biocontrol measure to block dengue virus (DENV) transmission (Moreira *et al.*, 2009; Ye *et al.*, 2015; Joshi *et al.*, 2017). Deeper knowledge of the interactions among the sand fly, its microbiota and the pathogens these insects transmit could lead to the discovery of new methods to block sand fly-transmitted diseases. Among alternative control strategies is paratransgenesis, where bacteria normally found in a specific insect is engineered to interfere with pathogen transmission (Coutinho-Abreu *et al.*, 2010*b*; Hurwitz *et al.*, 2011*a*). The first and crucial step in this approach is the identification of suitable commensal microorganisms in the vector. For safety reasons, these microorganisms should be non-pathogenic to man and other animals.

The search for candidates to paratransgenic blockade of *Leishmania* transmission by the kala-azar vector *Phlebotomus argentipes* identified two bacteria which met the above requirements, the commensals *Bacillus megaterium* and *Brevibacterium linens* (Hillesland *et al.*, 2008). More recently, the same group infected *P. argentipes* with a transgenic GFP expressing bacteria *Baccillus subtilis*, and demonstrated that the transduced bacteria was stably maintained in the *P. argentipes* gut (Hurwitz *et al.*, 2011*b*).

Along with the fact that the resident microbiota might establish a competitive or mutualistic interaction with acquired pathogens, insects possess an active immune response to balance and protect themselves from diseases and challenges that these microbes may cause. Insect immune responses are triggered through the recognition of evolutionarily conserved pathogen-associated molecular patterns (PAMPs) by host pattern recognition receptors (PRRs). The binding of PAMPs leads to the activation of defense mechanisms and pathways: RNAi, Janus-kinase/signal transducers and activators of transcription (JAK-STAT), Immune deficiency (IMD) and Toll, that will determine the production of effectors molecules such as antimicrobial peptides (AMPs) and reactive oxygen species (ROS) (Lemaitre *et al.*, 1995; Brennan and Anderson, 2004; Blair, 2011; Zeidler and Bausek, 2013). In addition to combating foreign invaders, components of the insect innate immune system are also involved in stress responses, wound healing and the management of microbial symbiont populations (Welchman *et al.*, 2009).

In the following text, we will address the complex interactions among sandflies, their microbiota and pathogens they transmit.

## Sand fly and viruses

Insects are hosts to a vast variety of viruses. Some viruses are unique to insects (reviewed in Vasilakis and Tesh, 2015; Roundy *et al.*, 2017), others (Arboviruses) are transmitted to other organisms, including animals and plants (reviewed in Blanc and Gutierrez, 2015; Ng and Zhou, 2015). Vector-borne viral diseases such as dengue, chikungunya and Zika are among the most devasting illnesses to afflict humanity.

Viral presence in insects such as mosquitoes and *Drosophila* elicits an antiviral immune response mediated by different mechanisms (e.g., Toll, IMD, JAK-STAT, etc.). Although each of these signalling pathways plays a specific role in the antiviral response (Kingsolver *et al.*, 2013; Merkling and van Rij, 2013; Xu and Cherry, 2014), the RNAi mechanism is reported to be the most active in insect antiviral response (Kemp *et al.*, 2013; Nayak *et al.*, 2013; Tassetto *et al.*, 2017). RNAi controls virus replication through the small non-coding RNAs called small interfering RNAs (siRNAs) in conjunction with an enzyme complex. These siRNAs associate with Argonaute (Ago) proteins to identify and destroy viral RNAs in a sequence-specific manner. Other eukaryotic small RNAs, such as microRNAs (miRNAs) and Piwi-interacting RNAs (piRNAs), which regulate cellular gene expression (reviewed in Asgari, 2013) and transposon activity (reviewed in Weick and Miska, 2014), have also been implicated in antiviral defense (Vijayendran *et al.*, 2013).

Although much is known about viral infections of mosquito vectors and the model organism *Drosophila*, much less is known about viral infections of sand flies, in which case, viruses can basically be considered as neglected pathogens (Depaquit *et al.*, 2010). Phleboviruses transmitted by sand flies have a relevant role as human pathogens (Alkan *et al.*, 2013). This genus comprises approximately 70 named viruses that are classified into two broad groups according to their antigenic, genomic and/or vectorial relationships: the sand fly fever virus group and the Uukuniemi-like virus group. The sand fly fever group includes Rift Valley fever virus (RVF) transmitted by mosquitoes and Toscana viruses (TOSV) transmitted by phlebotomine sand flies (Depaquit *et al.*, 2010). A large number of new sand fly-borne phleboviruses were recently described based on phlebovirus phylogeny reconstruction (Moriconi *et al.*, 2017).

In the Old World, at least 250 million people are exposed to Phlebovirus infections (Moriconi *et al.*, 2017). Sand fly fever Sicilian Viruses (SFSV) and TOSV, both transmitted by sand flies, are prominent human pathogens (Ayhan *et al.*, 2017). TOSV is an emerging pathogen and the cause of summer meningitis in the Mediterranean region, for which defined reservoirs were not identified. It is unlikely that humans are the reservoir for TOSV because human viremia is too short-lived (Depaquit *et al.*, 2010). Competent sand fly species might act as reservoirs in the viral cycle through transovarial transmission (Maroli *et al.*, 1993, 2013) since male sand flies were found to be infected by TOSV in nature.

Despite the fact that sand flies are proven vectors of leishmaniasis there are only a few reports describing the phlebotomine midgut infection by both *Leishmania* and a virus. Interestingly, one study showed that wild-caught and laboratory-reared *P. papatasi* infected with cytoplasmic polyhedrosis virus (CPVs) were refractory to experimental *Leishmania major* infection (Warburg and Ostrovska, 1987). CPVs cause a chronic pathology in the sand fly mid-gut that is characterized by structural abnormalities in the epithelium and the peritrophic matrix (PM) that interferes with blood digestion (Warburg and Ostrovska, 1987). These gut anomalies might hinder attachment to destroyed midgut epithelial cells and also lead to an early exposure of the parasites to sand fly digestive process and immune effector molecules, thus affecting *Leishmania* development.

In another study, sand flies trapped in an urban area of Marseille, France were infected with either *Leishmania* or phlebovirus. Curiously dual infections were not detected in this study, despite the local co-circulation of both pathogens (Faucher *et al.*, 2014). These two publications suggest an incompatibility between *Leishmania* and concomitant virus infection in the sand fly midgut. The complexity of *Leishmania*-sand fly-virus relationship is illustrated in another work where sand flies from Eastern Thrace and Northern Cyprus were analysed for presence of a virus and/or *Leishmania*. A pool of *Phlebotomus tobbi* was found co-infected by Toscana virus and *Leishmania infantum* (Ergunay *et al.*, 2014), implying different levels of coexistence events between various *Leishmania* and viruses. Further experiments should be performed with individual insects to confirm the coinfection hypothesis. These results reveal the complexity of the relationship between the *Leishmania* and viruses inside the sand fly mid-gut. More studies should be carried out to identify whether some of the different sand fly virus species could be used to control the *Leishmania* transmission. Much more is known about sand fly-transmitted viruses in the Old World than in the New World. The importance given to viruses transmitted by sand flies in Europe is due to the gravity of the disease they cause and high incidence in the local population (Depaquit *et al.*, 2010). On the other hand, there is little information about viruses transmitted by sand flies, or the diseases they cause, in the New World. Approximately 500 Phlebotominae species are known in the Americas, of which at least 56 are known to transmit leishmaniasis (Maroli *et al.*, 2013; Bates *et al.*, 2015). Comer *et al.* (1991) studied a New Jersey serotype (VSV-NJ) virus of the genus *Vesiculovirus* (family Rhabdoviridae), a causative agent of vesicular stomatitis in cattle, horses, and pigs (Comer *et al.*, 1991) on Ossabaw Island (Georgia, USA). In this study, the authors suggest that the vector for this virus was the phlebotomine sand fly *Lutzomyia shannoni*. Nunes-Neto *et al*. (2017) provided insights into the genetic diversity, classification and evolution of phleboviruses by characterizing six previously unclassified phleboviruses isolated in Brazil (Ambe, Anhanga, Joa, Uriurana, Urucuri and Tapara viruses) (Nunes-Neto *et al.*, 2017). Aguiar *et al.* (2015) developed an interesting approach to identify viral infections in different insects based in the production of viral small RNAs produced by host responses as exemplified by the RNA interference pathway (Aguiar *et al.*, 2015). The authors used the small RNA size profile unique signature to identify novel viruses. Using this method six novel viruses were identified in fruit flies, mosquitoes and sand flies. Among these, viruses named *Lutzomyia Piaui reovirus 1* (LPRV1) and *Lutzomyia Piaui reovirus 2* (LPRV2) and *Lutzomyia Piaui nodavirus* (LPNV) were found in *L. longipalpis*.

JAK-STAT is the classical pathway that responds to viral infections in mammals and this is also true for *Drosophila* (Arbouzova and Zeidler, 2006). In mosquitoes, the JAK-STAT pathway is also active against viruses, but the IMD and Toll pathways also play an important role against the infection in some specific mosquito–virus pairs (Ruckert *et al.*, 2014; Saraiva *et al.*, 2016).

Surprisingly, there is almost no information about sand fly responses to viral infections. The first report on sand fly immune responses to a virus infection was published by our group in 2008 when a non-specific antiviral response was identified in the *L. longipalpis* embryonic cell lineage LL5 (Pitaluga *et al.*, 2008). When these cells were transfected with any double-stranded RNAs, including the mimetic poly(I:C), they became resistant to infection with a West Nile Virus-Like Particle (VLP). A similar non-specific antiviral response elicited by dsRNA was also later identified in a shrimp, the honey bee and a bumble bee (Flenniken and Andino, 2013; Piot *et al.*, 2015; Brutscher *et al.*, 2017). Recently our group carried out an exoproteomics analysis of LL5 cells after transfection with dsRNA (Martins-da-Silva *et al.*, 2018). Among the secreted proteins positively modulated by dsRNA transfection, several were related to immunity and/or anti-viral response. Of special interest was the increased abundance of a phospholipid scramblase, which in mammals acts as an interferon-induced protein mediating antiviral activity.

The knowledge of the immune mechanisms involved in insect responses to the presence of viruses and other pathogens can provide important tools for the control of arboviral diseases that affect millions of people worldwide. In the case of sand flies, the role of resident microbiota in insect viral infections is still an open question. The identification of molecular agents that make an insect refractory to a specific virus is crucial for the development of novel control strategies.

## Sand fly microbiota

**Fig. 1.**
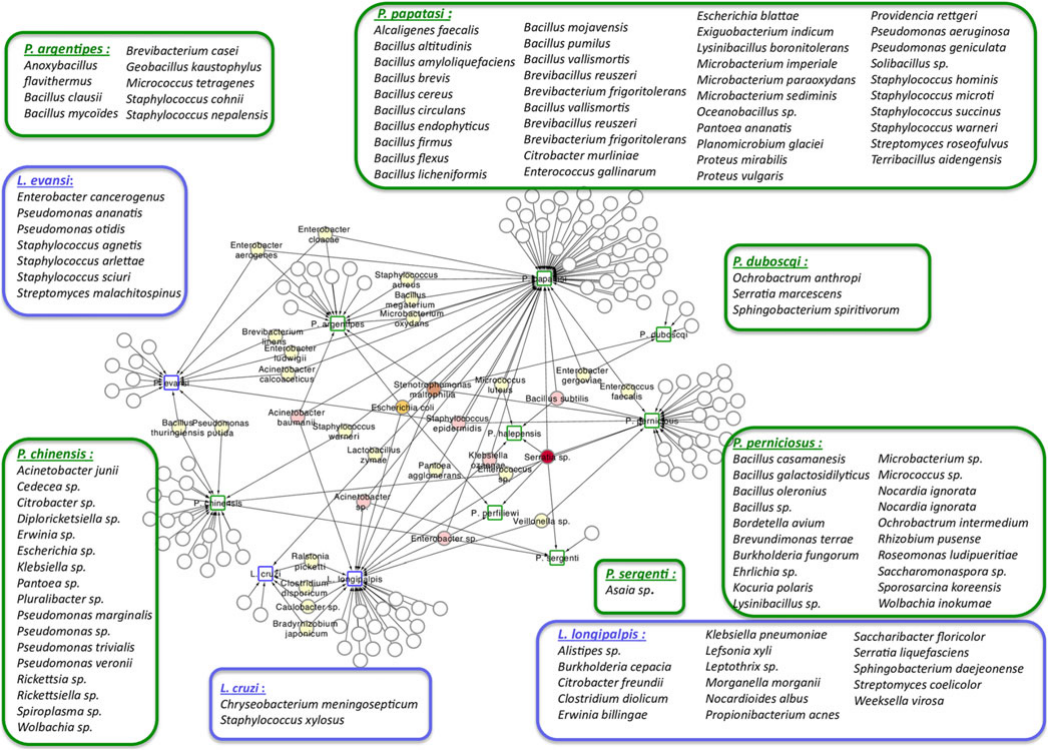
Network analysis showing the shared bacteria species found in sand flies. *Phlebotomus* sand flies are identified by squares surrounded by green and bacteria found in *Lutzomyia* sand flies identified with squares surrounded by blue. Coloured circles represent bacteria species that are shared between sand flies species. White circles represent bacteria species that are unique to each of the sand flies species and are listed inside large rectangles. The network representation suggests some relationships between the 11 studied New World and Old World sand fly species and the bacteria inhabiting their gut. *Bacillus thuringiensis* was isolated from *L. evansi* and *P. chinensis*, two sand fly species belonging to the New World and Old World, respectively (Fraihi *et al.*, 2017).

Phlebotomine sand flies lay their eggs in the soil, animal burrows or tree trunk niches, and larvae develop feeding on organic matter available in these sites (reviewed in Killick-Kendrick, 1999; Feliciangeli, 2004; Ready, 2013). The ingested food, together with environmental microbes, gives rise to the larval gut resident microbiota. The interest in the study of this microbiota is multifold, from obtaining basic information on how the vector responds to the presence of different microorganisms to how these interact with other pathogens, such as *Leishmania*. This knowledge may lead to the development of new strategies to control the spread of diseases, such as paratransgenesis. The importance of this subject led to the production of a large number of publications that are listed in Table 1. This list includes studies that describe microbiota obtained from multiple sources of sand fly samples (e.g. nature or laboratory), that use various technical approaches (e.g. culture or direct sequencing) to identify the resident microbes. In a recent publication focusing on the identification of resident microbiota of *Phlebotomus perniciosus* from the Western Mediterranean region, the authors performed a network analysis which suggests a pattern of interactions between sand flies and their microbiota (Fig. 1) (Fraihi *et al.*, 2017). The knowledge of the presence of a given bacterial species on various sand fly species might help in the development of paratransgenic bacteria targeting multiple vectors.

**Table 1.** Sand fly species with published gut microbiota data

Species	Developmental stage	Source	Country of origin	Identification technique	Microorganism	Reference
*P. duboscqi*	Larvae, pupae and adults	Colony	Senegal	Culturing	Bacteria	Volf *et al.* (2002)
*P. duboscqi*	Pupae and adults	Colony	Senegal	DNA sequencing	Bacteria	Guernaoui *et al.* (2011)
*P. papatasi*	Adults	Field	Egypt	culturing	Bacteria	Dillon *et al.* (1996)
*P. papatasi*	Adults	Field	Morocco	DNA sequencing	Bacteria	Guernaoui *et al.* (2011)
*P. papatasi*	Adults	Field	Iran	culturing	Bacteria and fungi	Akhoundi *et al.* (2012)
*P. papatasi*	Adults	Colony and field	Egypt, India, Tunisia and Turkey	Culturing and DNA sequencing	Bacteria	Mukhopadhyay *et al.* (2012)
*P. papatasi*	Adults	Field	Iran	DNA sequencing	Bacteria	Maleki-Ravasan *et al.* (2015)
*P. argentipes*	Adults	Field	India	Culturing and DNA sequencing	Bacteria	Hillesland *et al.* (2008)
*P. halepensis*	Adults	Field	Iran	Culturing	Bacteria and fungi	Akhoundi *et al.* (2012)
*P. kandelakii*	Adults	Field	Iran	Culturing	Bacteria and fungi	Akhoundi *et al.* (2012)
*P. perfiliewi*	Adults	Field	Iran	Culturing	Bacteria and fungi	Akhoundi *et al.* (2012)
*P. sergenti*	Adults	Field	Iran	Culturing	Bacteria and fungi	Akhoundi *et al.* (2012)
*P. chinensis*	Adults	Field	China	Culturing and DNA sequencing	Bacteria	Li *et al.* (2016)
*P. perniciosus*	Adults	Colony and field	Tunisia	Culturing and DNA sequencing	Bacteria	Fraihi *et al.* (2017)
*L. evansi*	Larvae, pupae and adults	Field	Colombia	Culturing and DNA sequencing	Bacteria	Vivero *et al.* (2016)
*L. longipalpis*	Adults	Field	Brazil	Culturing	Bacteria	Oliveira *et al.* (2000)
*L. longipalpis*	Adults	Colony	Brazil	Culturing	Bacteria	Perira *et al.* (2001)
*L. longipalpis*	Adults	Field	Brazil	Culturing	Bacteria	Gouveia *et al.* (2008)
*L. longipalpis*	Adults	Field	Argentina and Brazil	DNA sequencing	Bacteria and fungi	McCarthy *et al.* (2011)
*L. longipalpis*	Adults	Field	Brazil and Colombia	DNA sequencing	Bacteria	Sant'Anna *et al.* (2012)
*L. longipalpis*	Adults	Colony	Brazil	DNA sequencing	Bacteria	Kelly *et al.* (2017)
*L. longipalpis*	Adults	Field	Brazil	DNA sequencing	Bacteria and fungi	Pires *et al.* (2017)
*L. cruzi*	Adults	Field	Brazil	DNA sequencing	Bacteria	Sant'Anna *et al.* (2012)
*L. intermedia*	Adults	Field	Brazil	DNA sequencing	Bacteria	Monteiro *et al.* (2016)
*Nyssomyia neivai* (syn. *Lutzomyia neivai*)	Adults	Field	Brazil	DNA sequencing	Bacteria	Machado *et al.* (2014)

The microbial gut contents of colony-reared *Phlebotomus duboscqi* were investigated by using standard bacteriological methods to evaluate larvae, pupae and newly emerged insects. In the majority of analysed samples, *Ochrobactrum anthropi* was the dominant bacterium in all developmental stages, indicating the occurrence of bacterial transtadial passage (Volf *et al.*, 2002). Another study on colony-reared *P. duboscqi* used polymerase chain reaction-temperature gradient gel electrophoresis (TGGE) of the 16S rDNA gene fragment sequences obtained from different developmental stages (Guernaoui *et al.*, 2011). In this study, *Microbaterium* sp. was identified in immature and adult stages. This bacterium was previously identified by a soil bacterial consortium (Zhang *et al.*, 2007) indicating that the gut microbiota of immature sand fly stages can be influenced by external microbial populations. This leads to the idea that dispersion of a given microorganism in the larval environment in order to influence or manipulate these insects gut microbiota is a potential strategy for biological control of sand fly-vectored diseases. Indeed, sand fly larvae seem to prefer feeding on a nutrient- and microbe-rich food source mixed with the soil. In the laboratory, when different diets were offered to *Lutzomyia intermedia* and *L. longipalpis*, larvae from both species developed better when fed on nutrient-rich food composed of aged rabbit feces (Wermelinger and Zanuncio, 2001). Additionally, it has been suggested that dietary fungi are important to the development of *L. intermedia* based on pupation ratio (Wermelinger and Zanuncio, 2001).

The gut bacterial content of different developmental stages of *Lutzomyia evansi* from Central America was studied through culturing in different media and DNA sequencing of the resulting cultures (Vivero *et al.*, 2016). Identified bacteria across larvae, pupae and adults collected from the same locality included *Enterobacter*, *Pseudomonas*, *Bacillus* and *Lysobacter* genera (Vivero *et al.*, 2016). Interestingly, these bacterial genera are abundant in soil (Manfredi *et al.*, 2015; Thapa *et al.*, 2018). The presence of microbial strains in both larvae and adult sand flies will likely increase its utility in developing biological tools to control diseases transmitted by New World sand fly species.

In the same way that ingested food can influence gut microbiota in larvae, it can influence gut microbial content of adults as well. In nature male and female adult sand flies feed on carbohydrate-rich sources such as plant sap and aphid secretions (Wallbanks *et al.*, 1991; Anez *et al.*, 1994; Cameron *et al.*, 1995; Muller and Schlein, 2004), while females also feed on blood from birds and mammals, and in some cases, other vertebrates (Ghosh *et al.*, 1990; Mukhopadhyay and Ghosh, 1999; Afonso *et al.*, 2012; Brito *et al.*, 2014). Several reports focused on the gut microbial content of adult sand flies. Some of them used collected insect in the field, therefore submitted to a diverse diet, while other studies used insect from colonies that were fed on artificial defined diets.

The first report that identified the gut microbial content of New World adult sand flies used *L. longipalpis* collected from different localities in Brazil, or used insects obtained from a laboratory colony that had been artificially fed on blood or blood followed by sucrose solution (Oliveira *et al.*, 2000; Perira de Oliveira *et al.*, 2001; Gouveia *et al.*, 2008). From these studies environmental, gut-associated and opportunistic pathogenic species were identified including *Pantoea agglomerans*, *Stenotrophomonas maltophilia*, *Enterobacter cloacae*, *Pseudomonas* sp. and *Serratia marcescens*. These studies indicated that some genera are commonly shared between field and laboratory colony flies.

In addition to the applied culturing methods, *L. longipalpis* gut microbiota was investigated using denaturing gradient gel electrophoresis (DGGE) of 16S rDNA gene fragments amplified from insects collected at different localities in Brazil and Colombia (Sant'Anna *et al.*, 2012) or high-throughput metatranscriptome analysis using insects collected in Argentina and Brazil (McCarthy *et al.*, 2011). The culture independent-technique using DNA sequencing analysis considerably increased the microbiota detection range, therefore a larger number of bacterial species was identified. Below we will discuss the bacteria that are shared across several sand fly species.

**Fig. 2.**
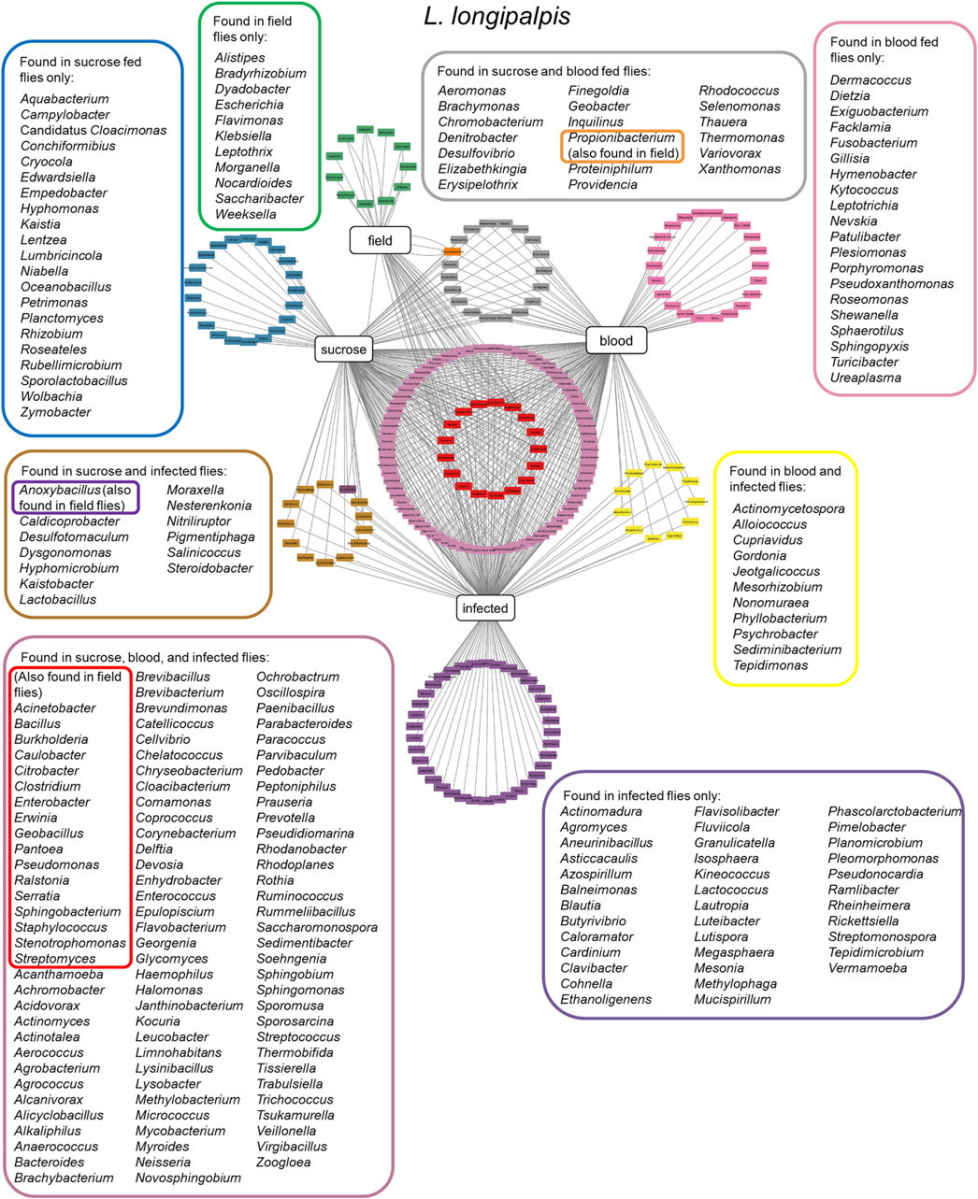
*Lutzomyia longipalpis* gut microbiota. Network analysis showing bacteria genera found in *L. longipalpis* obtained from field collections or laboratory-reared colonies. Feeding regimens are indicated: field (unknown feeding conditions), sucrose, blood, or infected by *Leishmania* (laboratory artificial feeding). Coloured border rectangles indicate bacteria genera found under each feeding regimen or shared between them. References used: Oliveira *et al.* (2000); Perira *et al.* (2001); Gouveia *et al.* (2008); McCarthy *et al.* (2011); Sant'Anna *et al.* (2012); Kelly *et al.* (2017); Pires *et al.* (2017).

More recently, two other studies were carried using colony-reared (Kelly *et al.*, 2017) or field (Pires *et al.*, 2017) *L. longipalpis* fed under laboratory conditions. Insects were fed on sucrose, blood or artificially infected by *Leishmania*, and microbial diversity was analysed by 16S or 18S rDNA sequencing. These studies demonstrated that microbial diversity decreased after blood feeding and that after blood digestion contents were eliminated the bacterial diversity recovered to previous sugar fed insect levels. A similar microbial ressurgence was found in *L. intermedia* (Monteiro *et al.*, 2016). The suggested idea of a quiescent resident microbiota present in non-fed females being altered by blood feeding and then returning in gravid females to a profile similar to non-fed females adds an interesting aspect to the microbial dynamics in the sand fly gut. It is important to mention that although bacterial diversity decreases after a blood meal, bacterial numbers actually increase. This might be due to some bacteria overgrowing others in a nutrient-rich environment (Volf *et al.*, 2002). Bacteria that are shared among *L. longipalpis* field and laboratory-reared insects (fed on sucrose, blood or *Leishmania* infected) (Fig. 2) belong mostly to the Proteobacteria phylum including *Pantoea*, *Serratia*, *Stenotrophomonas* and *Erwinia* genera. These are known to have an impact on *L. longipalpis* or other insects immunity (Boulanger *et al.*, 2004; Telleria *et al.*, 2013; Booth *et al.*, 2015; Heerman *et al.*, 2015; Husseneder *et al.*, 2017; Keita *et al.*, 2017). Other identified genera including *Acinetobacter*, *Burkolderia*, *Citrobacter*, *Enterobacter*, *Pseudomonas* and *Ralstonia* are commonly associated with *Phlebotomus*, mosquitoes, other insects or plants (Warburg, 1991; Dillon *et al.*, 1996; Eilmus and Heil, 2009; Akhoundi *et al.*, 2012; Maleki-Ravasan *et al.*, 2015; Lalithambika and Vani, 2016; Sun *et al.*, 2016; Husseneder *et al.*, 2017; Takeshita and Kikuchi, 2017; Thapa *et al.*, 2017; Osimani *et al.*, 2018; Song *et al.*, 2018; Ventura *et al.*, 2018). Interestingly, *Caulobacter* genus that comprises environmental associated microbes was found in *L. longipalpis* as well as in glassy-winged sharpshooter *Homalodisca vitripennis* (Rogers and Backus, 2014) but very little is known about this bacterium. The Firmicutes phylum, represented by the *Staphylococcus*, *Clostridium* and *Bacillus* genera, was found in *L. longipalpis* colony fed and field captured insects (Gupta *et al.*, 2014; Ngo *et al.*, 2016; Garofalo *et al.*, 2017). Bacteria from these genera are pathogenic to several organisms. There is a special interest in *Bacillus* species for potential sand fly biological control (Robert *et al.*, 1998; Wahba *et al.*, 1999). More specifically, *Bacillus subtilis* can colonize *L. longipalpis* larvae gut under laboratory conditions (Heerman *et al.*, 2015). Curiously the *Geobacillus* genus, that can form biofilms on food industry surfaces (Seale *et al.*, 2012; Al-Beloshei *et al.*, 2015) is present in the *L. longipalpis* studied samples and has been rarely studied in insects. The sand fly bacterial diversity and distribution indicates that although laboratory feeding systems can interfere in the *L. longipalpis* natural microbial diversity, some bacteria species can persist through the adult fly feeding and environmental differences (field and laboratory conditions). Moreover, the sand fly gut bacteria that are found in common in all the analysed conditions mentioned above are tightly associated with the insect environment, including plants. The sand fly plant-visiting habit for sap consumption (Petts *et al.*, 1997; Muller and Schlein, 2004) could be considered as a strategy to deliver microbial contents to manipulate the resident gut microbiota.

**Fig. 3.**
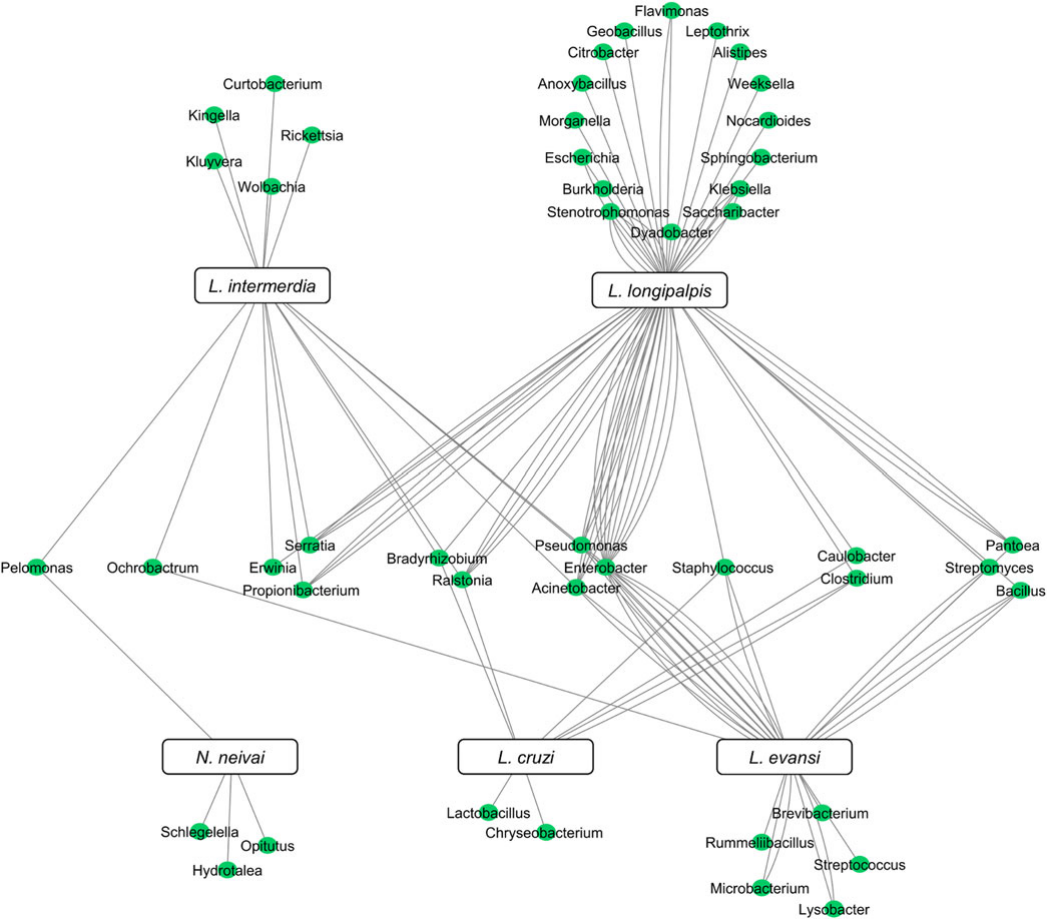
New World field sand flies microbiota. Network analysis showing bacteria genera found in *L. intermedia*, *L. longipalpis*, *L. evansi*, *L. cruzi*, and *N. neivai* (syn. *L. neivai*) obtained exclusively from field collection studies. References used: Oliveira *et al.* (2000); Gouveia *et al.* (2008); McCarthy *et al.* (2011); Sant'Anna *et al.* (2012); Machado *et al.* (2014); Monteiro *et al.* (2016); and Vivero *et al.* (2016).

Although the microbiota of field-collected and laboratory-reared *L. longipalpis* can have some bacteria species in common, the microbial diversity shared with other *Lutzomyia* species collected in the field can be quite reduced. To date, five New World sand fly species exclusively collected in the field had their microbial gut content investigated. Two of them are *L. evansi* (Vivero *et al.*, 2016) and *L. longipalpis* (Oliveira *et al.*, 2000; Gouveia *et al.*, 2008; McCarthy *et al.*, 2011; Sant'Anna *et al.*, 2012) mentioned above. Three other species were investigated using 16S rDNA sequencing or metatranscriptome: *Lutzomyia cruzi* (Sant'Anna *et al.*, 2012), *L. intermedia* (Monteiro *et al.*, 2016) and *Nyssomyia neivai* (synonymous *Lutzomyia neivai*) (Machado *et al.*, 2014). These field-collected sand fly species have very few shared bacteria (Fig. 3), most probably because they are exposed to diverse environments leading to diverse and distinct microbiota. Nevertheless, some shared species can be pointed out. *Pelomonas* sp., also found in other insects (Montoya-Porras *et al.*, 2018), was found in *N. neivai* and *L. intermedia*. Only *Ralstonia* sp. and *Bradyrhizobium japonicum*, present in other insects (Klimaszewski *et al.*, 2013; Rogers and Backus, 2014), were found in *L. cruzi*, *L. intermedia and L. longipalpis*. Among *L. evansi*, *L. intermedia and L. longipalpis* only three bacteria species were found in common: *Acinetobacter calcoaceticus* (found in other insect species, known for triggering a detectable immune response in tsetse flies) (Kaaya *et al.*, 1986; Hernandez-Flores *et al.*, 2015), *Enterobacter aerogenes* (found in other insects and potential pathogen to humans) (Memona *et al.*, 2017) and *Pseudomonas putida* (associated with soil and water) (Nicoletti *et al.*, 2015; Colauto *et al.*, 2016). *Staphylococcus agnetis* potentially pathogenic to poultry (Poulsen *et al.*, 2017) and associated to bovine mastitis (Lange *et al.*, 2015) was found only in *L. cruzi*, *L. evansi and L. longipalpis*.

In the Old World, *Phlebotomus papatasi* females from different collection sites had their microbial gut content investigated. Initial studies identified *Enterobacter cloaceae*, pathogentic to humans (Nagy *et al.*, 1998), from sand flies collected in Egypt (Dillon *et al.*, 1996) and *Microbacterium* sp., pathogenic to insects (Thakur *et al.*, 2015), from insects caught in Morocco (Guernaoui *et al.*, 2011). A larger number of bacteria species were identified from *P. papatasi* gut contents collected from Tunisia, Turkey and India, using culture brain heart infusion (BHI) medium followed by 16S rDNA sequencing. The majority of identified sequences belong to the *Bacillus* genus (Mukhopadhyay *et al.*, 2012) and depending on the culture media choice different bacteria could be identified. When comparing the microbial diversity of *P. papatasi* mentioned above with the data obtained through 16S ribosomal DNA sequencing collected in Iran (Maleki-Ravasan *et al.*, 2015) it is possible to point out some similar isolates such as *Acinetobacter*, *Enterobacter*, *Microbacterium*, *Staphylococcus* and *Terribacillus* genera. Additionally, other bacteria were identified at the species level such as *Bacillus cereus*, *Bacillus flexus*, *Bacillus licheniformis*, *Bacillus pumilus*, *B. subtilis*, *Pseudomonas aeruginosa* and *S. marcescens*.

Since sand fly environment and feeding habits can influence the sand fly microbial gut contents, one interesting study used 16S ribosomal DNA sequencing to investigate the bacteria present in the rodent *Rhombomys opimus* burrows where larvae feed on, *R. opimus* skin and intestinal track, as well as *P. papatasi* larvae and adult guts (Maleki-Ravasan *et al.*, 2015). *B. subtilis* and *Enterobacter cloacae* were identified in all analysed samples indicating that these two bacteria species can survive in *P. papatasi* environment, vertebrate host skin and gut, and sand fly gut. Moreover, these two bacteria are good candidates to be used in paratransgenic methods (Maleki-Ravasan *et al.*, 2015).

Other *Phlebotomus* species were investigated and their microbial content identified by microbiological methods. *Phlebotomus sergenti*, *Phlebotomus kandelakii*, *Phlebotomus perfiliewi* and *Phlebotomus halepensis* collected in Iran harbour several bacteria that were also found in *P. papatasi* such as *Acinetobacter*, *Enterobacter* and *Pseudomonas* genera, and more specifically *B. subtilis* and *S. marcescens* (Akhoundi *et al.*, 2012). *Acinetobacter* and *Bacillus* genera were also identified in *P. argentipes* collected in India through culturing and 16S rDNA sequencing (Hillesland *et al.*, 2008) supporting the potential use of bacteria from these genera in biological control strategies.

In China, the *Phlebotomus chinensis* associated microbial community presents interesting characteristics. The majority of bacteria identified by sequencing a 16S rDNA clone library obtained from the gut contents of adult females of this species were found to be from the families Coxiellaceae, Enterobacteriaceae mostly from the genus *Enterococcus*, and Pseudomonadaceae mostly from the genus *Pseudomonas*. Additionally, the intracellular *Diplorickettsia*, *Rickettsia*, *Rickettsiella*, *Spiroplasma* and *Wolbachia* were identified with these methods in the same study (Li *et al.*, 2016). Curiously, *Rickettsia* and *Wolbachia* were found only in sand flies collected from a locality where anthroponotic visceral leishmaniasis occurs and dogs and other animals are the reservoirs (Li *et al.*, 2016). *Diplorickettsia*, *Rickettsiella* and *Spiroplasma* were found only in sand flies collected from a locality where zoonotic visceral leishmaniasis occurs and humans are the identified reservoirs. It would be of interest to experimentally show the connection between these different microbiota profiles with vetorial capacity.

A study of the microbial gut content of *Phlebotomus perniciosus* collected in Tunis used culture-dependent and -independent techniques for bacteria identification (Fraihi *et al.*, 2017). When authors compared gut contents from field-caught and colony-reared insects they found that *Stenotrophomonas maltophilia*, *Bacillus* sp. and *Lysinibacillus* sp. are common to both groups of insects, suggesting that control strategies developed under laboratory conditions might be useful in the field (Fraihi *et al.*, 2017). In the same report, the authors show that variations in the resident microbiota are influenced by the sand fly species and particularities of niches where these insects live. Yet, an extensive meta-analysis showed an intricate network of several bacteria species associated with each of the sand fly species investigated and a relative small number of bacteria being shared among two or more sand fly species independent of the environment of harvest (Fig. 1). *Acinetobacter baumanii*, *E. coli*, *Stenotrophamonas maltophila*, *B. subtilis*, *Staphylococcus epidermidis*, *Acinetobacter* sp., *Enterobacter* sp., *Klebsiella ozaenae* and *Serratia* sp. are shared among at least three phlebotomine insects from Old and New World (Fraihi *et al.*, 2017).

While the gut resident bacteria of several sand fly species have been reported in multiple publication, there are limited reports on the fungal content of the sand fly gut. The presence of fungi species was reported in *L. longipalpis* guts collected from a non-endemic area for leishmaniasis. The fungal species identified by a high-throughput sequencing analysis were *Cunninghamella bertholletiae*, *Peronospora conglomerata*, *Mortierella verticillata* and *Toxicocladosporium irritans*, while no fungi sequences were found in the samples collected from an endemic area (McCarthy *et al.*, 2011) suggesting an excluding effect of fungi over *Leishmania* occurrence. On the other hand, when culturing techniques were used for isolating fungal contents of *P. papatasi*, *P. sergenti*, *P. kandelakii*, *P. perfiliewi* and *P. halepensis* collected in endemic areas of northern Iran, species belonging to *Penicillium*, *Aspergillus*, *Acremonium*, *Fusarium*, *Geotrichum* and *Candida* genera were identified (Schlein *et al.*, 1985; Akhoundi *et al.*, 2012), which are different fungi genus from those identified in *L. longipalpis*. Whether *L. longipalpis* resident fungi species have the potentials for controlling *Leishmania* presence is yet to be explored.

One of the main motivating forces for the study of vector microbiota is the possibility of identifying microorganisms potentially useful for the development of control strategies, as mentioned before. The data discussed above show the high complexity of microbiota in different sand flies and the difficulties of discussing the available data, considering the various and diverse methods of obtaining and analysing samples. In Fig. 2, we condensed these results using field or laboratory feeding parameters into a more comprehensible and friendly figurative display. What we can conclude is that some bacteria genera are regularly found across different sand flies, but at the species level there is less diversity. Thus, identifying a single bacteria that can be applied to control strategies targetted to a majority of sand fly vectors will be quite challenging. Additionally, when sand flies are kept in laboratory colonies they are able to host additional bacteria suggesting that these insects immune response is efficiently tunned to protect the insect integrity.

## Sand flies and bacteria: interdependence and immune responses

Recent research in many fields highlights the interdependence between many animals and their microbiomes. In the case of insect disease vectors, there is significant evidence showing the influence of resident non-pathogenic microorganisms on parasite–vector interactions.

The importance of bacteria in insect vector development had been demonstrated in mosquitoes as diverse as *Aedes aegypti*, *Anopheles gambiae* and the autogenous *Georgecraigius atropalpus* (Coon *et al.*, 2014). In all three mosquitoes, axenic larvae were unable to develop and the reintroduction of one single bacteria species was capable of rescuing development of *A. aegypti*. On the other hand, the complex interactions between microorganisms and disease-causing agents as viruses and *Plasmodium*, have been investigated. The review by Caragata and Walker (2012) covers the potential use of modified resident bacteria to fight pathogens and emphasizes the recent successful use of *Wolbachia* in controlling mosquito-borne diseases. The complex interactions of microbiota and mosquito vectors that go as far as influencing vector competence are also discussed (Caragata and Walker, 2012; Hegde *et al.*, 2015).

In the case of sand flies, bacteria seem to be important in many aspects of the flies’ life, starting with early development (Peterkova-Koci *et al.*, 2012). This study showed that *L. longipalpis* flies fed a diet containing raw rabbit feces were much more likely to lay eggs than flies fed feces that had been sterilized to remove all rabbit intestinal track-supplied bacteria. In addition, larvae fed on sterile feces had delayed hatching and lower survival rates. When different bacteria were reintroduced into sterile feces, there was a wide difference in hatching time and survival, demonstrating once again the importance of bacteria presence and specificity for insect development (Peterkova-Koci *et al.*, 2012).

Although breeding habits of sand flies in nature are still not clear, sand flies from tropical regions, like *L. longipalpis*, apparently breed in soil enriched with decomposed leaves and other detritus, with a preference for tree bases (Alencar *et al.*, 2011). Feeding habits of larvae in this environment are also scantily known, but evidence suggests a participation of microorganisms in the diet. In the laboratory, the direct feeding of larvae on fungi mycelia has been observed (Moraes *et al.*, 2012). Furthermore, the incorporation of different fluorescent bacteria to the diet and later detection of these fluorescent microorganisms in the gut proved the ingestion of these by the larvae. The presence of enzymes capable of digesting bacterial and fungal walls has also been found in insect guts confirming that environmental microbes could be a nutritional source for insects (Moraes *et al.*, 2012).

Although in most cases insects live at peace with their resident microbiota, this peaceful coexistence is the result of a complex balance between acceptance and rejection. Insects tend to mount immune responses to keep this balance. The expression of the anti-microbial peptide (AMP) defensin was investigated in *L. longipalpis* early stages of development (Telleria *et al.*, 2013). AMPs are small effector molecules involved in the innate immune response, composed of 5–100 amino acid residues, and found in plants and animals. AMPs are active against a broad spectrum of targets including viruses, bacteria, fungi and parasites (Bahar and Ren, 2013). Interestingly an increase of defensin expression was detected in late L4 larva stage, which stops eating in preparation for pupation and in pupae (Telleria *et al.*, 2013). Since it is well known that microbiota is practically abolished in pupal stages, the increased production of this defensin may be helping in clearing the insect gut of bacteria.

Further immunological studies in sand fly larvae involved the artificial introduction of Gram+ (*B. subtilis*) and Gram− (*P. agglomerans*) bacteria, normally present in the gut of *L. longipalpis* (Gouveia *et al.*, 2008), and the investigation of immune responses. A quite complex response was observed, with different outcomes related to Gram+ or −bacterial infections, and an apparent interplay between different immune pathways and effector molecules (Heerman *et al.*, 2015). One of the effectors investigated in this study was the negative IMD regulatory gene named *‘Poor immune response upon knock-in’* or *Pirk*. The expression of this gene was elevated at early times post infection (PI) with *P. agglomerans*, and this increased expression was maintained until 36 h PI. This might explain the downregulation of attacin at initial times PI. *B. subtilis* only affected the expression of *Pirk* at 24 h. On the other hand, IMD was upregulated only at 24 h PI with *P. agglomerans*, which might be responsible for an upregulated defensin expression at 24 h PI.

Immune responses to bacteria have also been reported in cultures of *L. longipalpis* LL5 embryonic cell line (Tesh and Modi, 1983). Insect cell lines have been widely used as a model to study vector immunity, being extensively exploited as a surrogate for understanding responses of mosquitoes to arbovirus infections (Walker *et al.*, 2014). Mosquito cells have also been employed in studies of insect immune response against bacteria, revealing the involvement of the Toll and IMD pathways upon exposure to both Gram+ and Gram− heat inactivated bacteria (Barletta *et al.*, 2012) in a way similar to what was observed previously in adult mosquitoes. With the advent of mosquito control approaches utilizing the bacteria *Wolbachia*, studies have been carried out to investigate mechanisms involved in the resistance to viral infection in mosquitoes harboring this endosymbiont, using both cell lines and insects (Rances *et al.*, 2012).

In the case of *L. longipalpis*, previous studies utilizing cell lines focused on the interaction of these cells with *Leishmania* (Rey *et al.*, 2000; Cortes *et al.*, 2011). Our group has investigated the response of LL5 cells to various organisms including viruses (Pitaluga *et al.*, 2008) as well as yeast, *Leishmania* and bacteria (Tinoco-Nunes *et al.*, 2016). LL5 challenges with the Gram− *Staphylococcus aureus* and the Gram+ *Escherichia coli* and *S. marcescens* activated the Toll and IMD pathways, with *S. aureus* and *S. marcescens* triggering an early response (Tinoco-Nunes *et al.*, 2016). AMPs seem to be under the control of different pathways, with cecropin and defensin 2 being under the control of the Toll and IMD pathways, the latter being produced as an early response to all challenges. Cecropin was shown to be produced early only in response to *S. marcescens* exposure, whereas the AMP attacin was produced at later times (Tinoco-Nunes *et al.*, 2016). *S. marcescens* is known to be pathogenic for *L. longipalpis* (Diaz-Albiter *et al.*, 2012), which might explain the early LL5 responses to this specific bacteria. These findings were important to establish LL5 cells as a reliable system to study *L. longipalpis* immunity.

The first report of putative immune responses of adult sand flies to bacteria came in 1997 when Nimmo *et al.*, detected anti-microbial molecules in the hemolymph of *L. longipalpis* previously injected with Gram+ or Gram− bacteria. This hemolymph showed lyzing properties against both *Micrococcus luteus* and *E. coli*, and specific bands were detected by gel electrophoresis, one of approximately 4kD which is compatible with the AMPs cecropin or defensin (Nimmo *et al.*, 1997).

A few years later the presence of AMPs was investigated in the Old World sand fly vector *P. duboscqi* (Boulanger *et al.*, 2004). In these studies, insects were exposed to the Gram− bacteria *Erwinia carotovora*, normally found in plants and in insect guts, initially by intrathoracic injection. The effect of this challenge was investigated by submitting insects’ hemolymph to HPLC where specific peptide peaks were identified. Two fractions harvested from HPLC analyses were found to have an antibacterial activity and after amino acid sequencing, one of them was identified as a defensin with high levels of similarity to mosquito defensins. This same defensin was also found in the gut of *P. duboscqi* after ingestion of *E. carotovora* (Boulanger *et al.*, 2004). Interestingly, this same study showed that this defensin was produced both in the hemolymph and in the gut of *P. duboscqi* following *L. major* infection, and the recombinant molecule was shown to be active against Gram− bacteria, yeast, fungi, and also *L. major*. This is initial evidence for a strong interplay between the production of immune molecules and their effect on bacteria and *Leishmania*, and the complexity of putative mechanisms involved in this interplay.

Further studies on the role of an AMP on sand fly bacterial infections were carried out in adult *L. longipalpis* by investigating the expression of a defensin in relation to infection with different bacteria and the route of bacteria acquisition on the outcome of immune responses. Insects were exposed to Gram– bacteria (*E. coli*, *Ochrobactrum* sp., *S. marcescens, P. agglomerans*) or the Gram+ bacteria *M. luteus*. When administered *per-os* all bacteria, with the exception of *P. agglomerans*, induced an increased transcription of the defensin gene with slight temporal differences depending on the microrganism the insects were fed on (Telleria *et al.*, 2013). Interestingly, among the four bacteria that produced an increased defensin gene expression, an earlier and stronger response was found in insects infected with *M. luteus*, an interesting finding since it has been reported that insect defensins are more effective against Gram+ bacteria (Nimmo *et al.*, 1997; Bulet *et al.*, 1999; Boulanger *et al.*, 2004). The inability to detect increased defensin expression in *L. longipalpis* fed on *P. agglomerans* is interesting in light of reports that this Gram− bacteria is a commensal found in the gut of many insects, and hence is considered a symbiont bacteria. In this same report, *L. longipalpis* was injected intrathoracically with *E. coli*, which brought a strong and lasting production of defensin. This is not unexpected, since the control injection itself brought a quite strong production of the AMP, most probably due to injury and the fact that microbiota is normally restricted to the insect gut and the presence of an intestinal bacteria in the hemolymph must be considered a main aggression by the sand fly, thus explaining the strong response.

Since the discovery of the Toll pathway in *Drosophila* (Lemaitre *et al.*, 1996), fruit flies have become well established as an excellent model to study immunity conferred by this pathway as well as other well-described pathways of insect immunity, IMD and JAK-STAT. Since then, the involvement of these pathways in immune responses of many insect vectors to parasites and viruses has been investigated (Cirimotich *et al.*, 2010; Sim *et al.*, 2014).

The involvement of the IMD pathway in *L. longipalpis* infection with *Leishmania* and bacteria was studied (Telleria *et al.*, 2012). In this report, the role of bacteria in controlling the expression of Caspar, a negative regulator of the IMD pathway, was documented in adult female flies using several different approaches. The report showed that when insects were treated with antibiotics, the expression of Caspar was increased in relation to untreated insects. This is consistent with a role of the IMD pathway in microbiota homeostasis since a decreased population of bacteria led to a higher Caspar expression resulting in a depressed IMD pathway. Also in this paper, the expression of Caspar was investigated in relation to *L. longipalpis* ingestion of exogenous bacteria. The expectation was that activation of the pathway would be associated with decreased expression of this negative regulator. This was seen when the insects were fed bacteria considered more pathogenic to insects: *M. luteus*, *E. coli* and *S. marcescens*, although, as expected, there were differences in the timing and extent of the decreased expression among the insects fed these pathogens. Interestingly, no increased expression in Caspar was observed with the symbiotic bacteria *P. agglomerans* and *Ochrobactrum* sp, indicating the insects’ immunological indifference to these harmless bacteria.

Other defense mechanisms, beyond the IMD pathway, have also been investigated in the bacteria–sand fly interaction. An important group of effector molecules are ROS, oxygen-derived radical species produced during cell respiration. ROS molecules include superoxide anion (O_2_^−^), the hydroxyl radical (OH^−^) and hydrogen peroxide (H_2_O_2_). ROS are involved both in defense against entomopathogens and in selection and control of commensal gut microbiota in various insects. In *Drosophila* ROS are produced in response to gut infections (Ha *et al.*, 2005), in *A. gambiae* ROS are produced following infection with the parasite *Plasmodium falciparum* (Molina-Cruz *et al.*, 2008) and also in *Anopheles aquasalis* infected with *Plasmodium vivax* (Bahia *et al.*, 2013). The involvement of ROS in bacterial control has been suggested from studies using *A. gambiae*. When these mosquitoes were treated with antioxidants, they were found to be more susceptible to bacterial infection than untreated insects (Molina-Cruz *et al.*, 2008). In the sand fly *L. longipalpis*, the involvement of ROS in responses to the pathogenic bacteria *S. marcescens* has also been investigated (Diaz-Albiter *et al.*, 2012). This study showed that the production of ROS was increased for up to 72 h post-infection in insects fed with the bacteria. Furthermore, the production of H_2_O_2_ was also found to be increased in *S. marcescens* fed flies. Feeding the insects with the ROS-scavenger uric acid caused premature *L. longipalpis* death that might be caused by a parallel increase of the native microbiota. On the other hand, under these circumstances *S. marcescens* numbers were decreased, which was interpreted by the authors as a consequence of the resident microbiota competition (Diaz-Albiter *et al.*, 2012).

Taken together, these studies reveal the complex interconnection of sand fly responses to bacteria that contribute to gut homeostasis. The importance of bacteria on the establishment of *Leishmania* infection in sand flies will be discussed below.

## Sand fly and *Leishmania*: a complex relationship

The *Leishmania* life-cycle in the insect begins when sand fly females ingest blood from an infected mammal. Within the sand fly, the acquired *Leishmania* develops exclusively inside the digestive tract (Sacks and Kamhawi, 2001). During their development, the parasites undergo changes to adapt to their new environment and develop into the form infective to another mammal host. In the process named metacyclogenesis, the parasites change from the intracellular spherical aflagellated amastigotes acquired from the ingested vertebrate blood cells to elongated flagellated, extracellular infective metacyclic forms. These morphological changes are accompanied by molecular modifications. For example, alterations on the surface of the parasite enable the interaction with the insect midgut, a fundamental step for parasite survival, development and subsequent infectivity to the vertebrate host (Bates, 2008).

To be successfully transmitted the parasite needs to overcome several obstacles, among which is infecting the correct sand fly species host. Among more than 900 species of sand flies recorded, just 98 are proven vectors of human leishmaniasis, 42 *Phlebotomus* species in the Old World and 56 *Lutzomyia* species in the New World (Volf and Myskova, 2007; Maroli *et al.*, 2013).

The sand fly vector competence depends on several factors such as a preference for feeding on humans, being infected with the *Leishmania* species occurring in humans and being able to complete their development inside the midgut after the blood meal digestion. In nature, living in sympatry is not equivalent to vector competence, since restrictive or specific vectors transmit only particular species of *Leishmania* (e.g. *P. papatasi* and *L. major*) (Sacks and Kamhawi, 2001). Other sand fly species are considered permissive or nonspecific, as they are able to harbour experimental infections of several *Leishmania* species (e.g. *L. longipalpis* and *Leishmania infantum chagasi* or *Leishmania mexicana*).

The success of sand fly midgut colonization by *Leishmania* is determined by several molecular factors. The first challenge of *Leishmania* within the sand fly is to resist the digestive process, which is achieved by interfering with the sand fly digestive enzymes activity (Dillon and Lane, 1993; Schlein and Jacobson, 1998; Sant'anna *et al.*, 2009; Telleria *et al.*, 2010) and modulating the transcription of several digestive enzymes genes (Ramalho-Ortigao *et al.*, 2007; Jochim *et al.*, 2008; Pitaluga *et al.*, 2009; Dostalova *et al.*, 2011). Furthermore, according to one publication, *Leishmania* secretes a myoinhibitory peptide that arrests hindgut peristalsis, thus delaying fecal elimination and increasing parasite persistence within the insect (Vaidyanathan, 2004). Also, *Leishmania* damages the insect stomodeal valve interfering in the blood ingestion process. These strategies facilitate parasite colonization of the midgut and increase the likelihood of subsequent transmission (Schlein *et al.*, 1992; Volf *et al.*, 2004). From the sand fly point of view, the *Leishmania* is an unwanted guest that causes indigestion, gut constipation and difficulties to swallow the blood meal.

A fundamental element in the sand fly digestive process is the PM, a semi-permeable membrane composed of glycoproteins associated with chitin fibrils which isolates the digestive bolus from the midgut epithelia and might have a role *Leishmania* midgut colonization (Hegedus *et al.*, 2009).

Studies with *P. papatasi* have demonstrated that the PM biogenesis homeostasis is fundamental for the parasite colonization success. The inhibition of the PM formation, by addition of an exogenous chitinase to the blood meal, resulted in sand flies refractory to *L. major* infection (Pimenta *et al.*, 1997). More recently it was suggested that *Leishmania* mortality is not caused directly by sand fly proteases and might result from toxic products of the blood meal digestion (Pruzinova *et al.*, 2018). Nevertheless, the interruption of the PM degradation through the silencing of an insect chitinase gene (Coutinho-Abreu *et al.*, 2010*a*), also resulted in refractoriness to *Leishmania* infection, indicating the need for more experiments approaching this subject.

Although in comparison with more aggressive parasites, such as *Plasmodium*, which transverses the mosquito gut and is exposed to the insect hemolymph, *Leishmania* can be considered a more mellow parasite, since it inhabits the sand fly gut in an apparently less aggressive fashion. Nevertheless, the evidence is mounting to show that this passage through the digestive system does not go unnoticed.

## Sand fly immunity to *Leishmania*: dealing with an unwanted passenger

In contrast to the many studies on other insect immune response to parasites, not much is known about the sand fly immune response to infections with *Leishmania*. As described above, insect immune responses lead to the stimulation of various immune mechanisms. Among these are RNAi, JAK-STAT, IMD and Toll, that will determine the production of effectors molecules (Lemaitre *et al.*, 1995; Brennan and Anderson, 2004; Blair, 2011; Zeidler and Bausek, 2013).

The sand fly's ability to counteract microbial infections has been demonstrated in many studies. In the case of parasite infections, the isolation and subsequent characterization of an active antimicrobial peptide defensin of *P. duboscqi* induced by *L. major* challenge was the first study demonstrating, at the molecular level, a sand fly humoral immune response elicited by *Leishmania* infection (Boulanger *et al.*, 2004). More recently the involvement of a *L. longipalpis* defensin in the sand fly immune response was investigated (Telleria *et al.*, 2013). In this report, *L. longipalpis* females were challenged with different bacteria species (discussed above) or with *L. mexicana* either orally or through microinjection. Interestingly, contrary to what was seen with *P. duboscqi*, the *L. longipalpis* oral *Leishmania* challenge did not stimulate the defensin transcription. It is possible that other *L. longipalpis* AMPs (not examined in this report), rather than this defensin, are induced by *L. mexicana* challenge. An interesting aspect of these different responses is the fact that *P. duboscqi* is an Old World restrictive vector, whereas the *L. longipalpis* is a New World permissive vector (Dostalova and Volf, 2012). While attachment to the gut in specific vectors (*P. papatasi*, *P. duboscqi* and *P. sergenti*) involves parasite lipophosphoglycan (LPG), this molecule is not required for parasite attachment in other sand fly species permissive for various New World *Leishmania* species, including *L. longipalpis* (Myskova *et al.*, 2007).

The lower induction of defensin in *P. duboscqi* challenge with *L. major* LPG defective mutants showed the involvement of the LPG molecule in the *P. duboscqi* immune response (Boulanger *et al.*, 2004).

Sand fly transcriptomic studies have reported changes in the expression of several immune-related genes in insects challenged with *Leishmania*, including components of the Toll, IMD and JNK pathways as well as oxidative stress-related molecules such as the antioxidants glutathione s-transferase, catalase, copper-zinc superoxide dismutase and peroxiredoxin, responsible to control ROS levels (Thannickal and Fanburg, 2000; Dillon *et al.*, 2006; Ramalho-Ortigao *et al.*, 2007; Pitaluga *et al.*, 2009; Abrudan *et al.*, 2013). Among genes related to the immune signaling pathways, an *L. longipalpis* Caspar gene was identified. The Caspar gene is a homologue of the human Fas-associating factor 1 protein, which negatively controls the IMD pathway (Kim *et al.*, 2006). The *L. longipalpis* Caspar gene expression profile has been recently investigated following challenge with bacteria (discussed above) and *Leishmania*. The effects of experimental Caspar RNAi silencing on the *Leishmania* infection success was also established. Importantly, *L. mexicana* challenge downregulated Caspar expression at the third and sixth days after infection while the silencing of Caspar led to a decreased *Leishmania* population size and infection prevalence (Telleria *et al.*, 2012). A role for the IMD pathway on parasite control was also seen in *Anopheles* sp. infected with *P. falciparum*, where the RNAi-mediated knockdown of Caspar reduced the protozoa survival in the insect (Garver *et al.*, 2009).

Insect immunity to *Leishmania* has also been investigated using insect cell lines. As mentioned earlier in this review, multiple reports have confirmed the immune competence of diverse insect cell lineages, including the LL5 embryonic cell line from *L. longipalpis*. Tinoco-Nunes *et al.* (2016) challenged LL5 cells with different microorganisms, including *L. i. chagasi*. The presence of *Leishmania* led to the upregulation of the Cactus gene, the negative regulator of the Toll pathway, whereas the expression of Caspar, the IMD pathway negative regulator, did not change significantly. The Dorsal and Relish genes, positive modulators of the Toll and IMD pathways, were upregulated by the *Leishmania* challenge and the expression of the AMPs attacin, cecropin and defensin 2 increased at different time points (Tinoco-Nunes *et al.*, 2016). These results revealed that the Toll and IMD pathways are involved in the sand fly LL5 cell line immune response against *Leishmania*.

Sand flies and mosquitoes present divergent oxidative responses to protozoan parasite infection. Whereas the presence of *Plasmodium* in the *Anopheles* gut produces an intense oxidative response with increased ROS production, *Leishmania* infected *L. longipalpis* do not show significant changes in ROS gut level when compared with control insects (Molina-Cruz *et al.*, 2008; Diaz-Albiter *et al.*, 2011). These opposite responses between sand flies and mosquitoes might be related to differences between the parasites more than differences between the insects hosts. Studies on macrophage infection by *Leishmania* revealed a direct relation between parasite virulence and antioxidant enzymes expression and cell ROS levels control, with the parasites lacking antioxidant enzymes being less virulent than normal parasites. The decrease of sand fly gut *Leishmania* population after silencing of the sand fly antioxidant enzyme catalase suggests that *Leishmania* oxidative escape also depends on manipulation of vector antioxidative elements (Pal *et al.*, 2010). The hypothesis is that *Leishmania* modulates the ROS levels in sand fly midgut during digestion, diminishing the activity of endogenous and exogenous antioxidant enzymes, to produce a friendlier developing environment.

Since *Leishmania* development occurs exclusively in the vector gut in the presence of a dynamic microbiota, our understanding of the parasite infection process inside the vector gut is challenged by multipartite factors. In the subsequent section, we discuss this aspect under the light of some recent and important publications.

## Sand fly, microbiota and *Leishmania*: an even more complex interrelation

*Leishmania* is not alone in the sand fly midgut. When the parasites reach the insect digestive tract they encounter the gut microbiota, a rich commensal microorganism community, with bacterial predominance that naturally colonizes the sand fly gut (discussed above).

As mentioned above, the insect microbiota plays important roles in vector physiology, such as nutrition and digestion (Dillon and Dillon, 2004) and can also act on the maturation of the innate immune system (Weiss *et al.*, 2011). The relationship between vector-borne pathogens and insect gut microbiota has been highlighted in several reports. The data produced suggest that microbiota can influence the parasite infection through the activation of vector innate immune pathways leading to induction of effectors molecules that will help in the control of infection by insect-vectored disease agents. As an example, the *L. longipalpis* midgut ROS suppression revealed the significant role of microbiota in facilitating *Leishmania* infection (Diaz-Albiter *et al.*, 2011).

Immune responses mounted against midgut bacteria in the mosquito and symbiotic bacteria in the tsetse fly have a protective effect against infections by insect-vectored viruses and parasites. Studies have shown that the mosquito employs some of the same immune factors to combat bacteria and *Plasmodium* parasite infection (Beier *et al.*, 1994; Dong *et al.*, 2006; Weiss *et al.*, 2013). Furhermore, *A. gambiae* previously treated with antibiotics to eliminate the midgut commensal bacteria were more susceptible to *Plasmodium* infection than untreated mosquitoes, and the reintroduction of various midgut bacteria restored the resistance phenotype (Dong *et al.*, 2009; Meister *et al.*, 2009).

The stimulation of tsetse flies antibacterial immune responses by the endosymbiont bacteria *Wigglesworthia* has been implicated in trypanosome control. The tsetse peptidoglycan recognition protein LB (PGRP-LB) induced by the presence of *Wigglesworthia* and effector molecules of the activated fly IMD pathway presented direct anti-trypanosome activity (Wang *et al.*, 2009). On the other hand, bacteria may also directly inhibit pathogen development, either by disrupting necessary interactions between the pathogen and vector epithelium or through the production of anti-parasite molecules (Azambuja *et al.*, 2005). An *Enterobacter* bacterium isolated from wild *Anopheles* populations in Zambia was found to decrease *Plasmodium* development through the production of ROS, suggesting that a mosquito-mounted response was not required for the observed infection inhibition (Cirimotich *et al.*, 2011).

As seen in other vector–parasite interaction models, the microbiota influences the sand fly vector competence, although existing work presents some divergent results. In experiments with colony-raised *L. longipalpis,* pre-feeding the insects with the bacteria *Asaia* sp., *Ochrobactrum intermedium* and a yeast-like fungus *Pseudozyma* sp. previously isolated from wild-caught and laboratory-reared female *L. longipalpis*, prevented *Leishmania* establishment in the sand fly midgut (Sant'Anna *et al.*, 2014). In the same work, the authors verified that *L. longipalpis* previously infected with *L. mexicana* were resistant to *Serratia* infection, thus identifying a protective effect of *Leishmania* infection against *Serratia* challenge. These results pointed to a competitive relationship between *Leishmania* and microbiota.

Recently this interpretation was revised in two papers, both demonstrating that *Leishmania* development in the vector is dependent on microbiota. Firstly, the presence of bacterial phylogenies in midguts of sugar-fed, blood-fed and *L. infantum*-infected colony-reared *L. longipalpis* was studied. Also, the effect of antibiotics treatment on *Leishmania* infection was investigated. The results of these studies showed that while *Leishmania* infection led to an escalating loss of bacterial diversity throughout the course of infection, the depletion of *L. longipalpis* midgut microbiota after antibiotics treatment impaired *L. infantum* replication and development to infective metacyclic forms (Kelly *et al.*, 2017). The second work, conduced by Louradour and collaborators (2017), carried out experiments of *L. major* infection in *P. duboscqi* under various conditions. As observed in Kelly and collaborators, treatment with antibiotics prevented the development of *Leishmania* in the midgut of *P. duboscqi*, and feeding with bacterial culture supernatants was not able to reverse the antibiotic effect. The introduction of engineered antibiotic-resistant bacteria isolated from natural *P. duboscqi* microbiota to the midgut did not impair the *Leishmania* infection as reported in Sant'Anna *et al.* (2014). Differences in the number of time points assessed and differences in the number of bacteria used in these studies might explain these different outcomes. In the work by Kelly *et al*. (2017) the authors used a high concentrations of bacteria (>10^7^ CFU mL^−1^) and investigated only one early time point, while Lauradour and collaborators (2017) employed low bacterial doses (10^4^ CFU mL^−1^) and investigated diverse time points throughout the *Leishmania* development. Experiments with sand flies treated with antibiotics and fed with different sugar meal concentrations showed that sugar concentrations usually used to maintain the insects (15–30%) impaired *L. major* growth. These latter results suggest that the role of microbiota in the sand fly and *Leishmania* interaction might be to ‘buffer’ the ‘milleu’ osmolarity.

The complexity of insect vector, protozoan pathogen and microbiota relationship can be observed in another recent work where evidence was presented for the involvement of microbiota in the *Anopheles* PM formation. Mosquitoes treated with antibiotics showed a reduction in the expression of several genes related to the PM production. In addition, microscopy revealed disruptions in the PM integrity in antibiotic-treated insects (Rodgers *et al.*, 2017). The potential participation of sand fly PM in the establishment of *Leishmania* in the sand fly midgut was already addressed above. The possibility that the phenomenon is seen in *Anopheles* also occurs in other insect models raises doubts about the interpretation of the results associating pathogen infection and microbiota depletion after antibiotic administration. Disruptions in the sand fly PM integrity could permit the access of sand fly molecules damaging to *Leishmania*, before the parasite has undergone changes that enable resistance against the sand fly midgut hostile environment, leading to parasite losses.

The relevance of sand fly microbiota studies was reinforced in a recent work where the authors identified a crucial role for sand fly microbiota in *Leishmania donovani* infection in mammals. Dey *et al.* (2018) demonstrated that during the *L. longipalpis* blood meal microorganisms from vector microbiota are co-egested with *Leishmania* (Dey *et al.*, 2018). These microbes activate the mouse neutrophil inflammasome leading to a rapid production of interleukin-1b (IL-1b), which sustains neutrophil infiltration. These neutrophils help shield *L. donovani* parasites and promote infection of macrophages post transmission. The sand fly dysbiosis using antibiotic treatment impaired the *L. donovani* infection. This outcome suggests that the relationship between the *Leishmania* and the sand fly microbiota may go beyond the vector midgut, with the microorganisms presence modulating vertebrate host immune response, by producing a friendly ‘milleu’ fundamental for the success of the vertebrate infection by the transmitted parasite.

The information compiled here reveals that there is a complex relationship among the sand fly, *Leishmania* and commensal microbiota. Greater understanding of the relationship and the mechanism that underpin it could identify targets for the development of strategies to control the ability of the sand fly to transmit leishmaniasis to man.

### Concluding remarks

In this review, we compiled and discussed the hitherto published data about the interactions among sand flies, its microbiota and sand fly-borne pathogens. Exploring available information we can say that although sand flies are important vectors of viruses in the Old World, in the New World there are still few studies on the putative sand fly vectorial competence for viral transmission. Strikingly, in sharp contrast to how much is known in mosquito–virus interactions, there is almost total absence of data on sand fly-virus interplay, pointing to the need of more studies to help understand pathways that could be involved in sand fly antiviral immune responses.

Another important point is the increasing awareness of the importance of microorganisms for the survival and development of sand flies, and for a successful parasite–vector interaction. The sand flies microbiota prospective studies disclosed the existence of a rich and variable intestinal flora. The recent growing data amassed by all these studies is a first step for the potential establishment of paratransgenesis strategies.

Since sand flies are mostly known as the vectors for leishmaniasis, a few studies have approached the question of how the insect responds to the parasite infection. Some data on the involvement of the IMD pathway, or the production of effector molecules are discussed herein, but much remains to be uncovered.

Very recent work has focused on how resident microbiota can affect the *Leishmania* infection of the vector with surprising results. Basically, it was found that the sand fly microbiota is fundamental for *Leishmania* development and transmission. One paper suggests that the removal of the microbiota alters the osmolarity of the intestinal environment and is thus deleterious for the *Leishmania* development. Since we do not know exactly which molecular mechanisms are responsible for this dependence of *Leishmania* on microbiota, this may be considered an open field of research. Interestingly, the *Leishmania*-microbiota relationship does not seem restricted to the sand fly midgut. Microorganisms egested during the bite together with *Leishmania* elicit an immune response that increases the *Leishmania* infectivity in mice.

The reviewed studies demonstrate the complex and intricate network among sand flies, microbiota, virus and *Leishmania.* More studies are needed to increase the knowledge about these very interesting interplays. This information will be crucial for the development of control and eradication measures of sand fly-borne diseases.
